# Successful management of dexmedetomidine for postoperative intensive care sedation in a patient with anti-NMDA receptor encephalitis: a case report and animal experiment

**DOI:** 10.1186/s40064-016-3079-3

**Published:** 2016-08-22

**Authors:** Daiki Yamanaka, Takashi Kawano, Hiroki Tateiwa, Hideki Iwata, Fabricio M. Locatelli, Masataka Yokoyama

**Affiliations:** Department of Anesthesiology and Intensive Care Medicine, Kochi Medical School, Kohasu, Oko-cho, Nankoku, Kochi 783-8505 Japan

**Keywords:** Dexmedetomidine, NMDA receptor, Encephalitis

## Abstract

**Background:**

Anti-*N*-methyl-d-aspartate receptor (NMDA-R) encephalitis is a recently identified but increasingly recognized autoimmune paraneoplastic disease. Because these patients present complex neuropsychiatric symptoms due to NMDA-R dysfunction, the optimal methods of sedation/anesthesia remain controversial. Here, we present animal experiment data, along with a related case report, implying the safe and effective use of dexmedetomidine in patients with anti-NMDA-R encephalitis.

**Findings:**

(1) Animal experiment: in order to investigate whether dexmedetomidine may interfere with NMDA-R activity, an NMDA antagonist (MK-801) model in rats was used to simulate anti-NMDA-R encephalitis. Administration of MK-801 produced well-characterized schizophrenia-like behaviors, i.e. hyperlocomotion and stereotyped sniffing. Ketamine, an NMDA receptor-dependent anesthetic, exaggerated both behaviors, even at sub-anesthetic doses. On the other hand, dexmedetomidine did not show any exacerbation, suggesting that dexmedetomidine has no clinically relevant interaction with the NMDA-R in vivo. (2) Case report: our patient, a 27-year-old female, was diagnosed with anti-NMDA-R encephalitis secondary to ovarian teratoma. She underwent laparoscopic ovariectomy under general anesthesia using thiopental, sevoflurane, and remifentanil, which were well tolerated. After transfer to the intensive care unit, she became increasingly agitated despite repeated boluses of intravenous fentanyl. Infusion of dexmedetomidine (0.5–1.0 μg/kg/h) was started, and an adequate level of sedation was achieved uneventfully. After discontinuation of dexmedetomidine, recovery from sedation was smooth and quick without any deterioration of neurological or psychological symptoms.

**Conclusions:**

Our experimental findings and the presented case suggest that dexmedetomidine may be safely used in patients with anti-NMDA-R encephalitis. Further clinical evaluation is warranted to validate this finding.

## Background

Anti-*N*-methyl-d-aspartate receptor (NMDA-R) encephalitis is an autoimmune disease first described in 2007 (Dalmau et al. 2011; Peery et al. 2012; Titulaer et al. 2013). It has since been increasingly recognized as one of the more common identifiable causes of encephalitis. These patients typically present with neuropsychiatric syndromes, including cognitive impairment, seizures, loss of consciousness, central hypoventilation, and autonomic nerve dysfunction. Its development is driven by an autoimmune reaction primarily against the NMDA-R. Anti-NMDA-R encephalitis is frequently a paraneoplastic syndrome; a recent multi-institutional observational study has shown that 38 % of patients have presence of a tumor, of which 94 % were ovarian teratomas (Dalmau et al. 2011). Initial therapy includes high-dose steroids, immunoglobulin, and plasma exchange, as well as removal of any causative neoplasm if present. Therefore, during the acute phase, most patients may undergo a surgical procedure and intensive care support, thus requiring general anesthesia and sedation. However, the optimal anesthetics/sedation methods for patients with anti-NMDA-R encephalitis remain to be established (Pryzbylkowski et al. 2011; Kawano et al. 2011; Lapébie et al. 2014; Broderick et al. 2014; Simon 2014).

The NMDA-Rs play a crucial role in controlling synaptic plasticity and memory function (Paoletti et al. 2013). The functional NMDA-R is composed of both the NR1 and NR2 subunits. The development of anti-NMDA-R encephalitis is associated with antibodies against NR1/NR2 heteromers, which reduce NMDA-R density by an increase in receptor movement into the plasma membrane, i.e., internalization (Hughes et al. 2010; van Coevorden-Hameete et al. 2014). The NMDA-Rs are also considered to be an important target of the most frequently used inhaled and non-inhaled anesthetics that inhibit its functions (Franks 2008). Therefore, sedative/anesthetic agents that act via antagonism of the NMDA-R may potentially aggravate the disease symptoms (Pryzbylkowski et al. 2011; Simon 2014).

Dexmedetomidine is now widely used to provide sedation, analgesia, and anti-sympathetic effects without respiratory depression during the perioperative period (Carollo et al. 2008). Additionally, recent studies suggest that dexmedetomidine exerts neuroprotective effects against brain injury in the central nervous system (Ma et al. 2004; Degos et al. 2013). The major sedative and antinociceptive effects of dexmedetomidine are due to its stimulation of α_2_-adrenoceptors located in the locus coeruleus (Hunter et al. 1997). Therefore, dexmedetomidine may be suitable for postoperative sedation of patients with anti-NMDA-R encephalitis. However, the interaction between dexmedetomidine and NMDA-R activity remains under investigation, and there have been no reports on the clinical use of dexmedetomidine in the patients with anti-NMDA-R encephalitis.

In this report, we present experimental data regarding the use of dexmedetomidine under simulated NMDA-R hypo-functions in rats and a clinical case of anti-NMDA-R encephalitis successfully sedated with dexmedetomidine postoperatively.

### Animal experiments

It is well reported that antagonists of the NMDA-R, such as phencyclidine, produce symptoms similar to those observed in patients with anti-NMDA-R encephalitis (Dalmau et al. 2011). Furthermore, an NMDA-R antagonist, MK-801, induces schizophrenia-like symptoms and is widely used as a rodent model of schizophrenia (Andiné et al. 1999). Using this animal model, we investigated whether dexmedetomidine can be safely used under NMDA-R-deficient conditions, a mimic of anti-NMDA-R encephalitis.

All procedures were approved by the Kochi University Animal Experiment Committee. For the rat model of psychosis, male Sprague–Dawley rats (6 weeks old, body weight: 145–180 g) were intraperitoneally (i.p.) injected with MK-801 (Sigma-Aldrich, St. Louis, MO). Injections of 0.01, 0.02, or 0.05 mg/kg dexmedetomidine (n = 8 for each dose) were tested in this study. The NMDA-R antagonist anesthetic, ketamine, was used as a positive control at doses of 10, 50, and 100 mg/kg (n = 2–8, as indicated in Results).

**Fig. 1:**
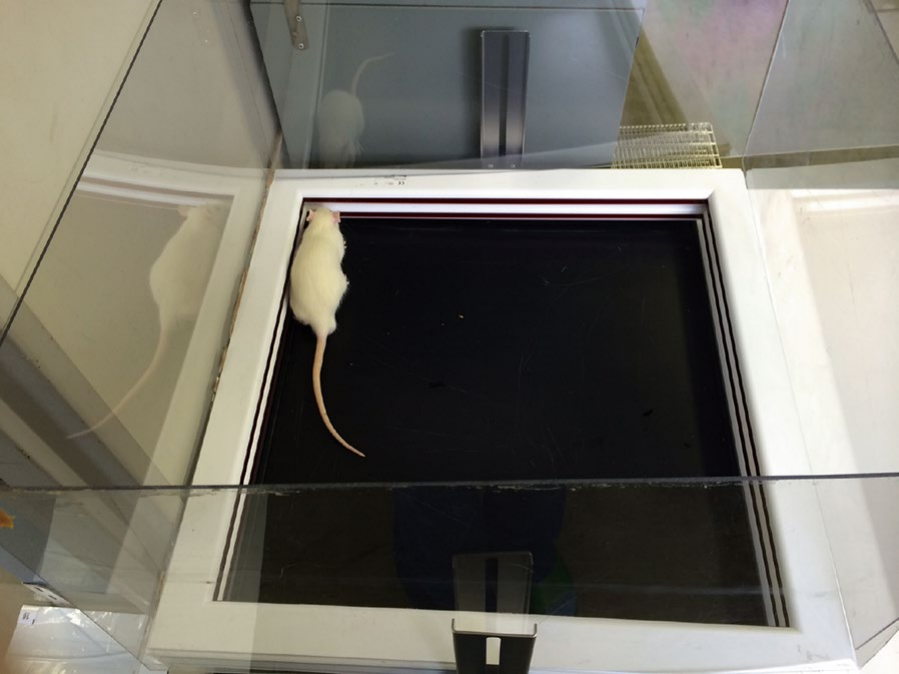
Picture of a rat during open-field test

On the day of the experiment, animals were placed inside a transparent Plexiglas chamber (45 × 45 × 40 cm). All animals were individually habituated in the test environment for at least 2 h prior to the test. MK-801 (0.1 mg/kg dissolved in 0.9 % saline) or an equal volume of saline was administered i.p., followed by a behavioral observation and rating procedure that started 15 min after the injection and continued for 60 min. Two types of behavior, locomotor activity and stereotyped behavior, which are well-characterized components associated with NMDA-R antagonism (Andiné et al. 1999), were assessed by an observer blind to drug treatment. Locomotor activity was measured using pairs of 16 photo beams positioned 10 cm apart and 5 cm from the floor of the cage (Fig. 1). The total count of horizontal beam crosses was recorded every 5 min. For stereotyped sniffing rating, animals received a score of 0 (absence), 1 (some rare), 2 (discontinuous, free interval of more than 5 s), or 3 (continuous), according to the method described previously (Sukhanov et al. 2014). The rating procedure consisted of observation for 30 s every 5 min. The results are presented as the mean ± standard error of the mean (SEM). Data were analyzed by two-way ANOVA repeated measures, followed by Bonferroni’s post hoc test. A *p* value of 0.05 was considered statistically significant.

**Fig. 2:**
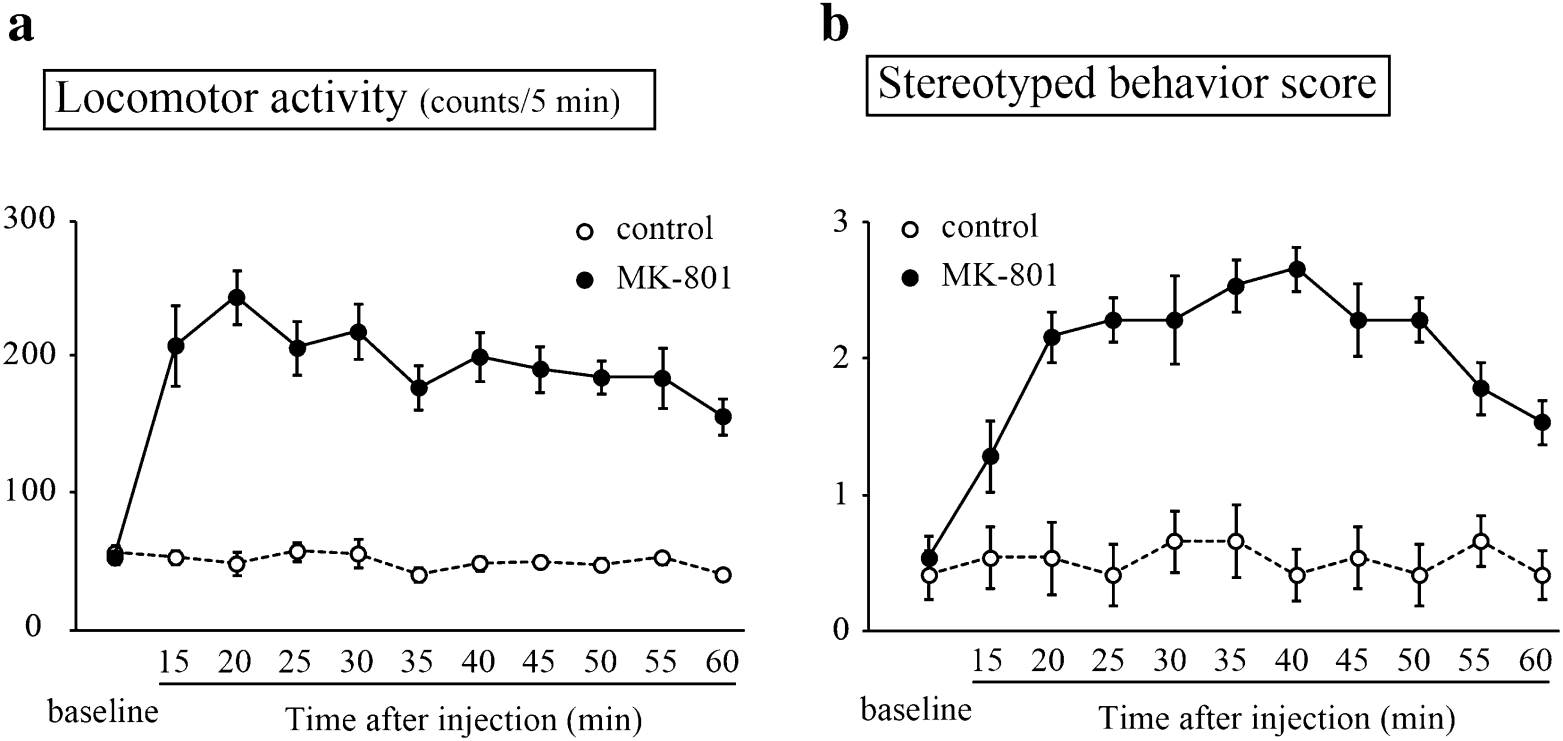
Time course of MK-801- or vehicle-induced locomotor activity (**a**) and stereotyped behavior (**b**). Each parameter was scored at baseline and at 5-min intervals starting 15 min after drug injection, and continued for 60 min. *Each vertical bar* represents the mean ± SEM (*n* = 8 in each experimental group)

**Fig. 3:**
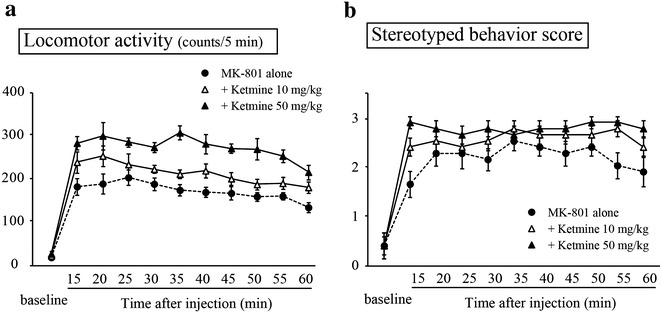
Effects of ketamine on MK-801-induced locomotor activity (**a**) stereotyped behavior (**b**). Each parameter was scored at baseline and at 10-min intervals starting 15 min after drug injection, and continued for 60 min after injection of either MK-801 alone or combined with ketamine (10 or 50 mg/kg). *Each vertical bar* represents the mean ± SEM (*n* = 8 in each experimental group)

**Fig. 4:**
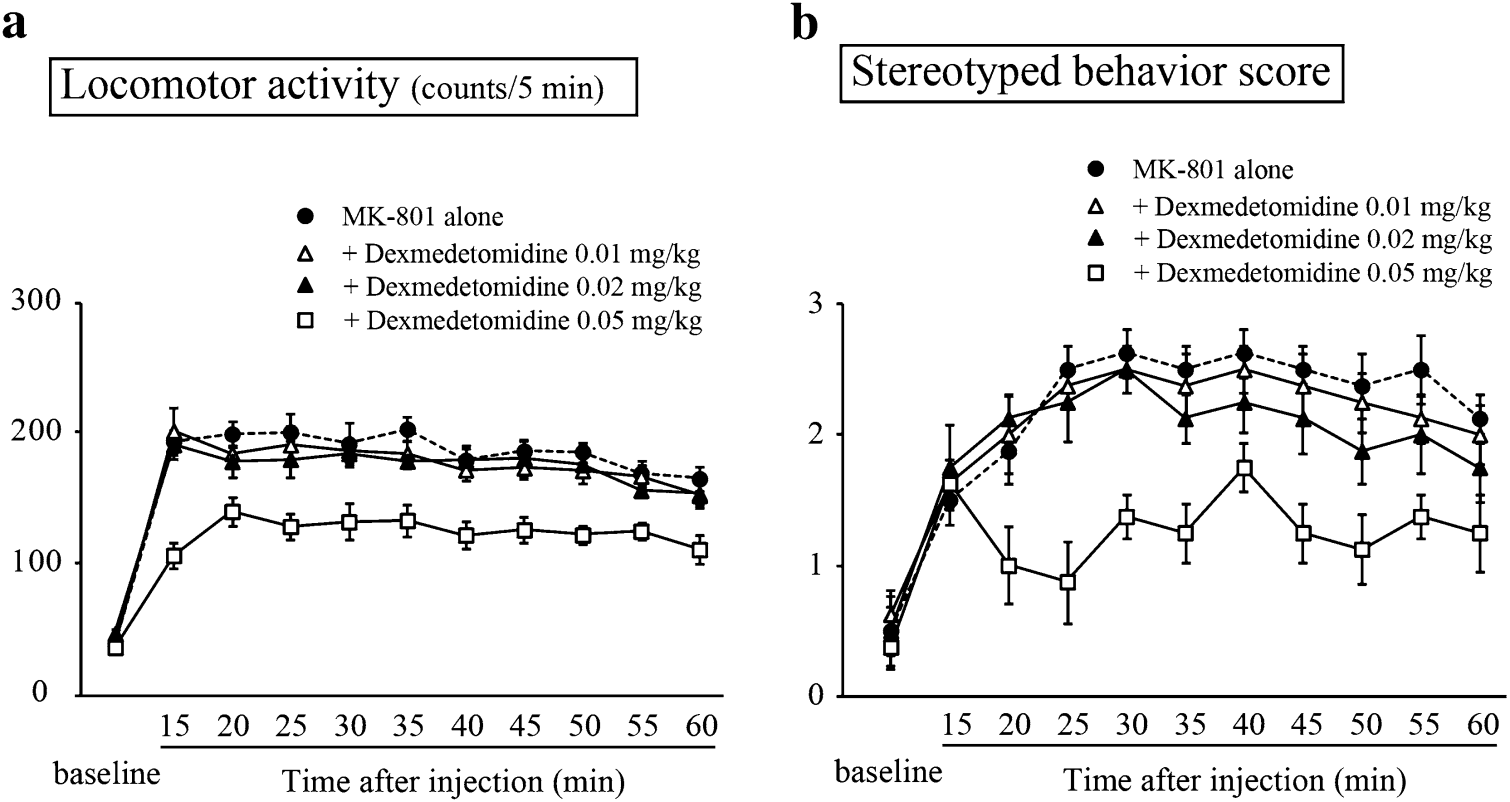
Effects of dexmedetomidine on MK-801-induced locomotor activity (**a**) stereotyped behavior (**b**). Each parameter was scored at baseline and at 10-min intervals starting 15 min after drug injection, and continued for 60 min after injection of either MK-801 alone or combined with dexmedetomidine (0.01, 0.02, or 0.05 mg/kg). *Each vertical bar* represents the mean ± SEM (*n* = 8 in each experimental group)

As with previous reports (Andiné et al. 1999), acute administration of MK-801 induced hyperlocomotion (Fig. 2a) and stereotypic sniffing (Fig. 2b), compared with control rats (*p* < 0.05, main effect of group for each variable). Sub-anesthetic doses of ketamine (10 and 50 mg/kg) dose-dependently exaggerated MK-801-induced hyperlocomotion (Fig. 3a) and stereotypic sniffing (Fig. 3b; both *p* < 0.05). For the anesthetic dose of ketamine (100 mg/kg), the first two rats tested transiently developed seizure-like movement, preventing appropriate behavioral assessment, and subsequent investigation at this dose was discontinued. These results offer preclinical proof-of-concept that NMDA-R antagonist anesthetics, such as ketamine and N_2_O, should be avoided in patients with anti-NMDA-R encephalitis, due to possible further deterioration of NMDA-R hypofunction. On the other hand, sub-sedative doses of dexmedetomidine (0.01 and 0.02 mg/kg) had no influence on MK-801-induced hyperlocomotion (Fig. 4a) and stereotypic sniffing (Fig. 4b). Furthermore, the sedative dose of dexmedetomidine (0.05 mg/kg) significantly attenuated both behaviors; this may have been associated with its sedative effect.

## Case report

**Fig. 5:**
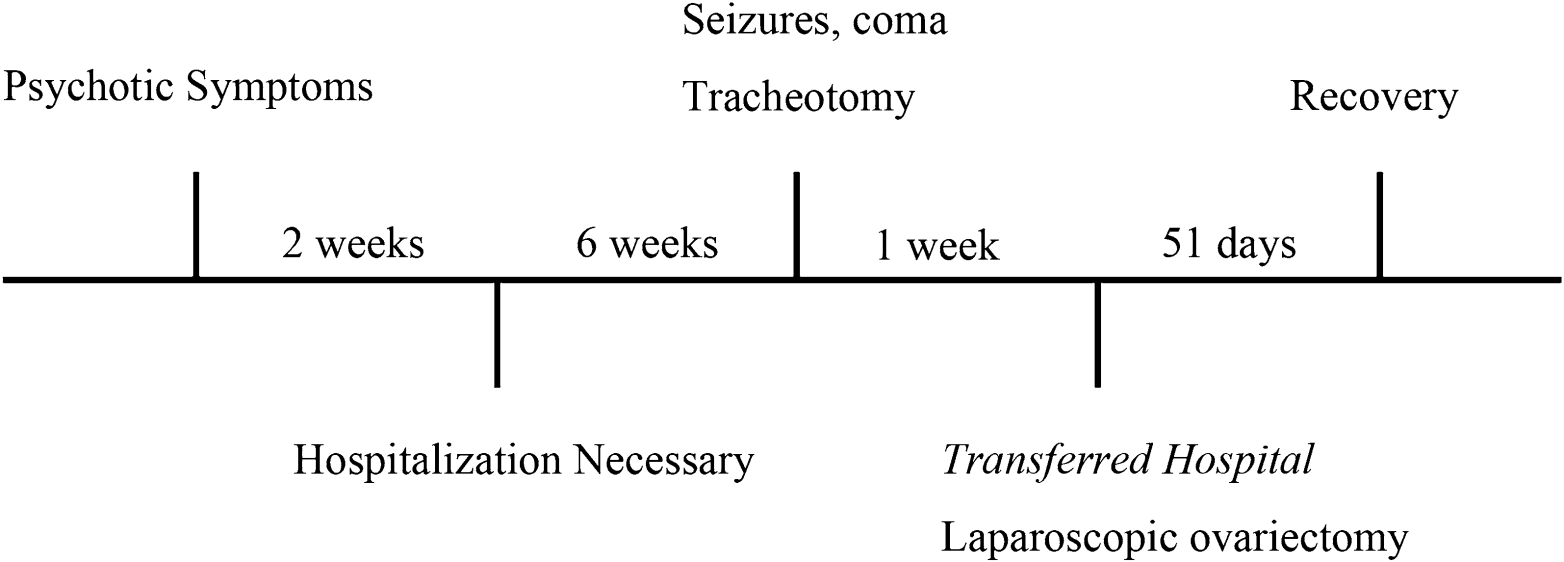
The patient’s trajectory of symptom presentation

A 27-year-old female with neither a history of psychiatric disorder nor mental retardation presented to her district general hospital with a 2-week history of headache, fever, slurred speech, and confused thinking. A timeline of the patient’s symptom trajectory is provided in Fig. 5. An electroencephalogram showed generalized non-specific slowing, and brain CT and MRI were normal. She was initially treated with acyclovir for possible viral encephalitis due to herpes simplex virus (HSV). However, the patient did not respond to the treatment, and developed progressive confusion, short-term memory dysfunction, and partial complex seizures 6 weeks later. To manage these symptoms, a single dose of midazolam (5 mg, IV) was administered, but the patient subsequently developed coma with a Glasgow coma score of 5 (eye opening 1, motor response 3, verbal response 1), and stayed unconscious for 3 days. During this period, the patient required tracheotomy and artificial ventilation. Repeated brain imaging was normal; however, anti-NMDA-R antibodies, but not HSV DNA, were detected in the cerebrospinal fluid. Subsequent lower abdominal MRI revealed a 3.5-cm left ovarian tumor, and the patient was diagnosed with anti-NMDA-R encephalitis. She was treated with plasma exchange followed by intravenous immunoglobulin without improvement, and was then transferred to our hospital for laparoscopic ovariectomy.

On the day of surgery, the patient underwent general anesthesia with endotracheal intubation, consisting of induction with thiopental, fentanyl, and rocuronium, followed by maintenance with sevoflurane (1.5–2.0 %) and remifentanil infusion. Surgery was uneventful and completed in 84 min, and the pathology confirmed mature teratoma. Consistent with a previous case report in Japan (Kawano et al. 2011), our case demonstrated that sevoflurane could maintain anesthesia without complication. Seven minutes after discontinuation of sevoflurane at the end of surgery, responses to verbal commands and spontaneous respiration via the tracheostomy cannula were observed, and she was transferred to the intensive care unit (ICU). However, the patient condition worsened with agitated, oral-lingual-facial dyskinesia, limb dystonia, as well as developed tachycardia and hypertension. Analgesia was attempted with repeated boluses of 50 µg fentanyl (total of 4 times), but failed to improve her symptoms. Dexmedetomidine was then infused at 1.0 μg/kg/h for the first hour, followed by maintenance infusion of 0.5 μg/kg/h. In this case, we chose to avoid the loading dose of dexmedetomidine to prevent any potential side-effects such as hypertension. An adequate level of sedation (Ramsay sedation score ≥3) was achieved without supplemental sedatives or analgesics. Upon sedation, hemodynamics and arterial blood gasses were stabilized. The day after surgery, the patient was responsive to verbal stimuli 15 min after discontinuation of dexmedetomidine infusion. The following postoperative course was uncomplicated, and she was discharged from the ICU on postoperative day 3. The patient showed steady clinical improvement, and was discharged from the hospital on postoperative day 51, with near complete recovery to her premorbid state.

## Discussion

Anti-NMDA receptor encephalitis is now thought to be caused by the disruption of NMDA function by anti-NMDA autoantibodies (Dalmau et al. 2011; Peery et al. 2012; Titulaer et al. 2013). It is frequently a paraneoplastic syndrome and surgical removal of tumor is associated with better outcomes. Since the most common used anesthetics/sedatives interact with NMDA receptor, it’s suspected that these anesthetics/sedatives leads to worsen the disease symptoms. More recent case reports indicated that general anesthesia with propofol or sevoflurane may be well tolerated (Pryzbylkowski et al. 2011; Kawano et al. 2011; Lapébie et al. 2014; Broderick et al. 2014; Simon 2014). On the other hand, there have been no reports concerning postoperative intensive care sedation of patients with anti-NMDA-R encephalitis. In this report, we first described a single case showing that dexmedetomidine may contribute to improve the symptoms of anti-NMDA-R encephalitis exaggerated after surgery while we cannot rule out the possibility that this symptoms were also associated with postoperative delirium. Nevertheless, our findings imply that dexmedetomidine is safe and suitable for postoperative sedation, and allows stable hemodynamic responses in patients with anti-NMDA-R encephalitis.

The data of animal experiment suggest that dexmedetomidine has no clinically relevant interaction with the NMDA-R in vivo. These results may provide mechanistic support for the present case, i.e., a sedative dose of dexmedetomidine may be safely used in patients with anti-NMDA-R encephalitis. In addition, the sedative dose of dexmedetomidine could attenuated MK-801-induced psychotic behavior (Fig. 4a, b). Consistent with our observations, it has been reported that clonidine, another clinically available α_2_ adrenergic agonist, could protect against MK-801-induced neurotoxicity (vacuole reaction) in rat retrosplenial cortex (Farber et al. 2002). Furthermore, the most significant side-effects of dexmedetomidine are hypotension and bradycardia (Carollo et al. 2008). However, these were not observed in the present case, and postoperative autonomic hyperactivity as well as confusion could be controlled. Taking all together, our findings suggest dexmedetomidine may be a better choice for patients with anti-NMDA-R encephalitis. Nevertheless, it is difficult to determine whether these effects were a direct pharmacological interaction of the dexmedetomidine or a secondary result of sedation. Therefore, further clinical studies including case studies and treatment experience are necessary to confirm this point of view.

In addition to dexmedetomidine, sedatives commonly used in the ICU include propofol and benzodiazepines. Propofol predominantly acts via direct activation of γ-aminobutyric acid (GABA) receptors, but also weakly inhibits NMDA-R (Lapébie et al. 2014; Simon 2014). There has been one case report of a worsening of neuropsychiatric symptoms including dyskinesias and tonic–clonic generalized seizure, while no adverse consequences occurred during its intra-operative use (Lapébie et al. 2014). Further research is needed to determine what effects, if any, propofol administration may have on the symptoms of anti-NMDA-R encephalitis. Benzodiazepines bind to specific sites on postsynaptic GABA receptors, and can help manage agitation and insomnia associated with anti-NMDA-R encephalitis (Kruse et al. 2014). However, although it is unknown whether benzodiazepines were responsible for this unexpected serious coma in the present case, sustained consciousness disturbance was observed after a one single sedative dose of i.v. midazolam, a short-acting benzodiazepine. Benzodiazepines are metabolized by hepatic microsomal cytochrome P450 (CYP) enzymes, specifically CYP3A4 (Olkkola and Ahonen 2008). Therefore, we cannot rule out the possibility that our patient was a CYP3A4 poor metabolizer, resulting in increased plasma concentrations of midazolam.

In conclusion, we report for the first time successful management of a case of anti-NMDA-R encephalitis with intravenous dexmedetomidine infusion for postoperative intensive care sedation. Preclinical data using a rat model also suggested that clinically relevant doses of dexmedetomidine had no noticeable effects on neurological or psychiatric symptoms associated with NMDA-R dysfunction. Our findings imply that dexmedetomidine can be safely used as a sedative agent for patients with anti-NMDA-R encephalitis.
